# Supplementary material for: Evaluation of Vaccine Strains Developed for Efficient, Broad-Range Protection against Foot-and-Mouth Disease Type O
Source: Vaccines (Basel). 2023 Jan 27;11(2):271. doi: 10.3390/vaccines11020271 (PMC9963059; doi:10.3390/vaccines11020271)
Supplement: Supplementary file 1 [file vaccines-11-00271-s001.zip › vaccines-2160183-supplementary.pdf]

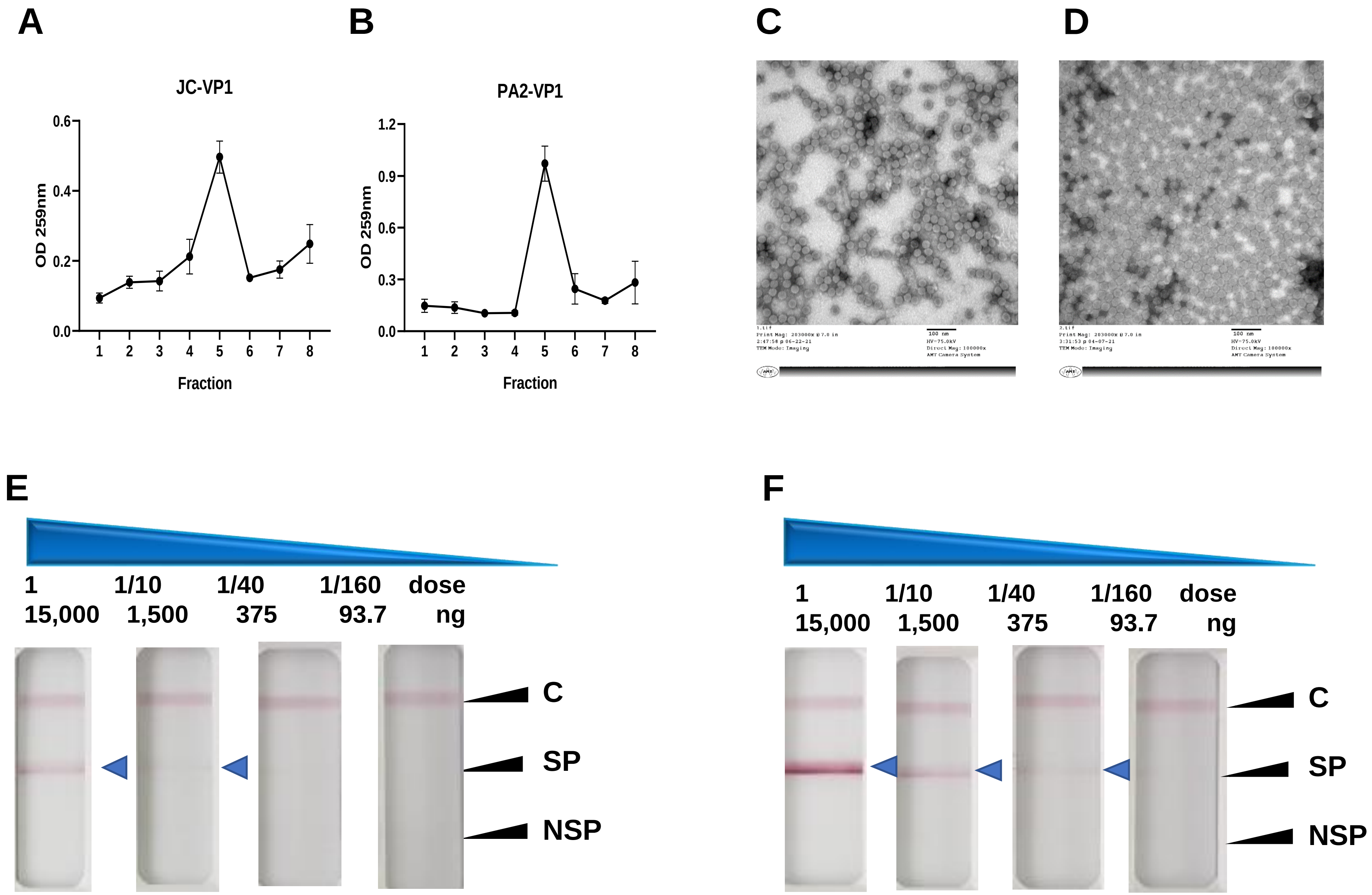

Figure S1. Vertification of Inactivated antigen, JC-VP1 and PA2-VP1.
(A, B) Spectrophotometer analysis of inactivated antigen, JC-VP1(A) or PA2-VP1(B) in 15%–45% sucrose gradient
(C, D) Transmission electron microscopy of inactivated antigens, JC-VP1(C) or PA2-VP1(D). The bar represents 100nm.
(E, F) Confirmation of inactivated FMDV antigen, JC-VP1(E) or PA2-VP1(F) dilution using lateral flow assay (PBM co. ltd) kit

Table S1. Comparison of critical antigenic residues in structural proteins for FMDV type O.

Strains	Site 1 (VP1)				Site 2 (VP2)													Site 3 (VP1)		Site 4 (VP3)									Site 5 (VP1)
	144	148	150	208	70	71	72	73	74	75	77	78	129	131	189	190	191	43	44	56	58	65	68	116	131	134	195	196	149
O Manisa	V	L	V	P	V	T	S	D	P	F	R	C	L	S	V	N	S	T	P	H	E	T	T	D	E	K	D	G	Q
O PA2-VP1	-	-	-	-	-	-	-	-	-	-	-	-	-	-	-	-	-	-	-	-	-	-	-	-	-	-	-	-	-
O JC-VP1	-	-	-	-	-	-	-	-	-	-	-	-	-	-	-	-	-	-	-	-	-	-	-	-	-	-	-	-	-
O BE	-	-	-	-	-	-	-	-	-	-	-	-	-	-	-	-	N	-	-	-	-	-	-	-	-	-	-	-	-
O JC	-	-	-	-	-	-	-	-	-	-	-	-	-	-	-	-	T	-	-	-	D	-	-	-	-	-	E	-	-
O TWN	-	-	-	-	-	-	-	-	-	-	-	-	-	-	-	-	-	K	-	-	D	-	-	-	-	-	-	-	-
O PA2	-	-	-	-	-	-	-	-	-	-	-	-	-	-	-	-	T	-	-	-	-	-	-	-	-	R	-	-	-

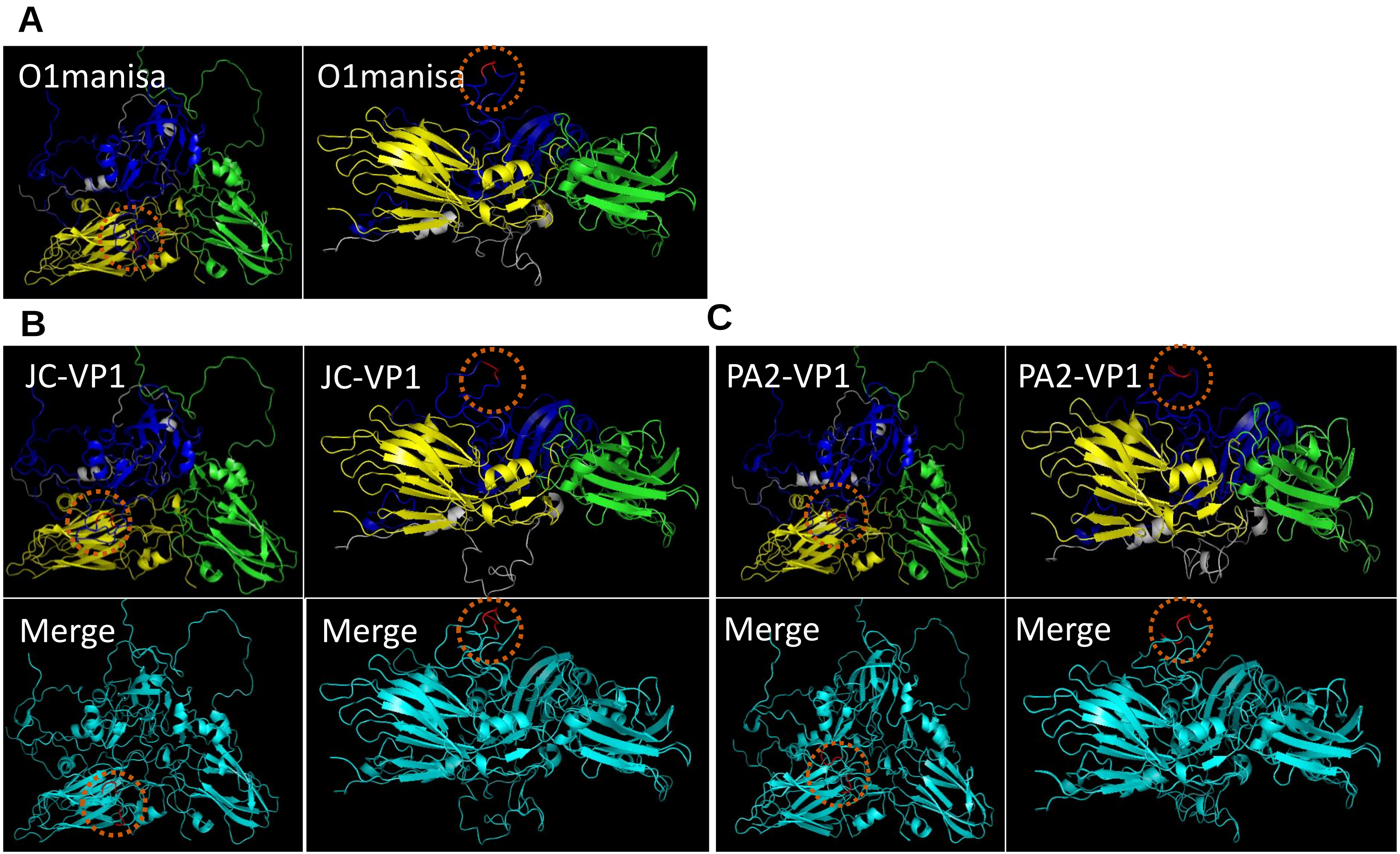

Figure S2. Schematic diagram of FMDV genomes with VP1 epitope addition for broad spectrum vaccine strains for type O.

(A) Surface structure of O1 manisa virus as a control

(B) Surface structure of JC-VP1

(C) Surface structure of PA2-VP1

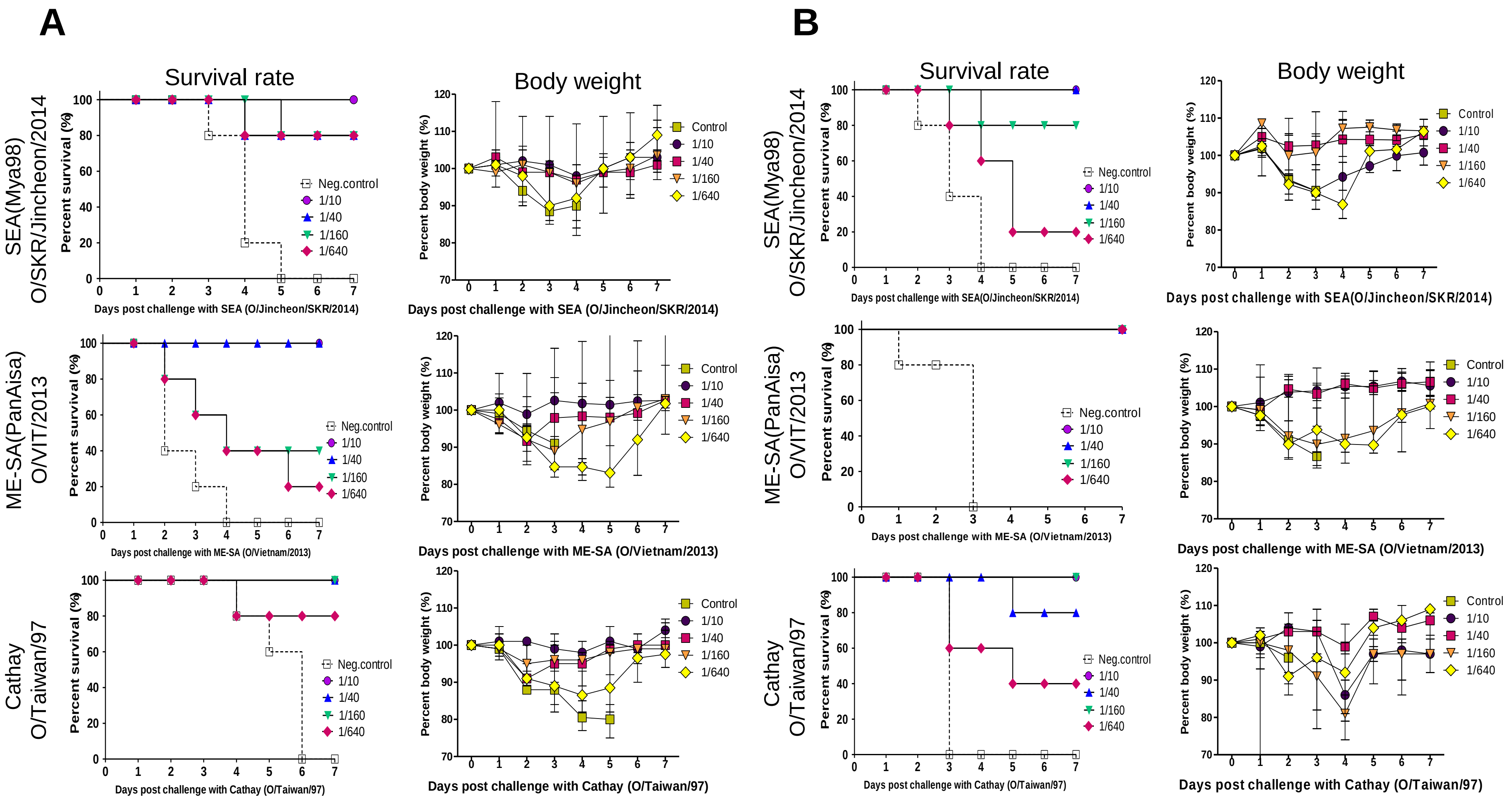

Figure S3. Protection of the vaccinated mice challenged with typical three topotypes of FMDV.

C57BL/6 female mice were vaccinated with 1/10(1.5μg), 1/40, 1/160, 1/640 dose of JC-VP1 (A) or PA2-VP1 (B) used for this experiment. The challenged viruses belong to topotype of ME-SA, SEA, and Cathay topotypes.

A

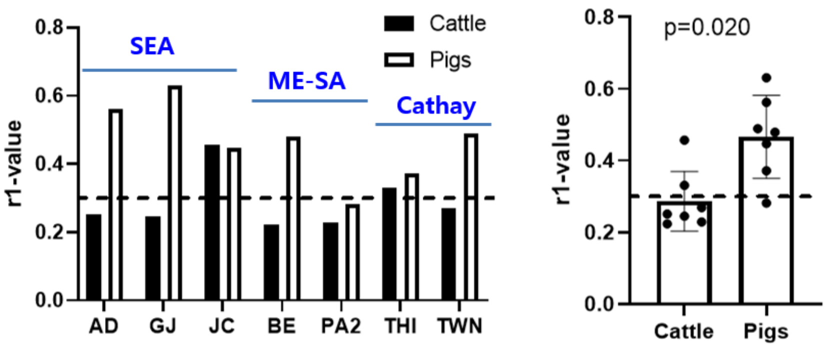

B

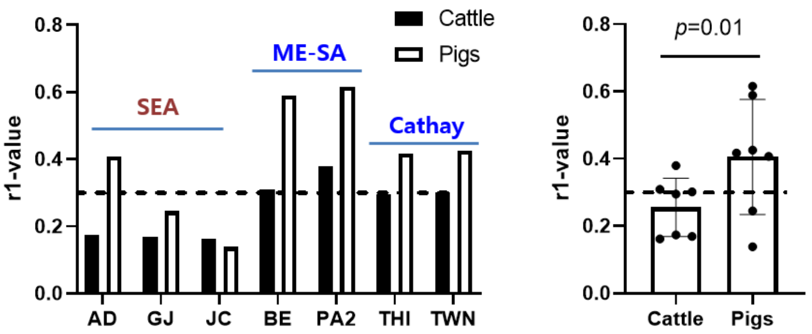

C

Sera from vaccinated animals / Topotype, strain		Cattle		Pigs	
		PA2-VP1	JC-VP1	PA2-VP1	JC-VP1
SEA	AD/2010	0.174	0.251	**0.407**	**0.562**
	GJ/2016	0.169	0.245	0.245	**0.631**
	JC/2014	0.162	**0.457**	0.138	**0.447**
ME-SA	BE/2017	**0.309**	0.224	**0.589**	**0.479**
	PA2/2008	**0.380**	0.229	**0.616**	0.282
Cathay	THI/1960	0.295	**0.331**	**0.417**	**0.372**
	TWN/1997	**0.302**	0.269	**0.426**	**0.489**

Figure S4. R1 value of JC-VP1 and PA2-VP1 in cattle and pigs.
(A) r1 value of JC-VP1 in cattle and pigs
(B) r1 value of PA2-VP1 in cattle and pigs
(C) Comparison of r1 value between JC-VP1 and PA2-VP1.

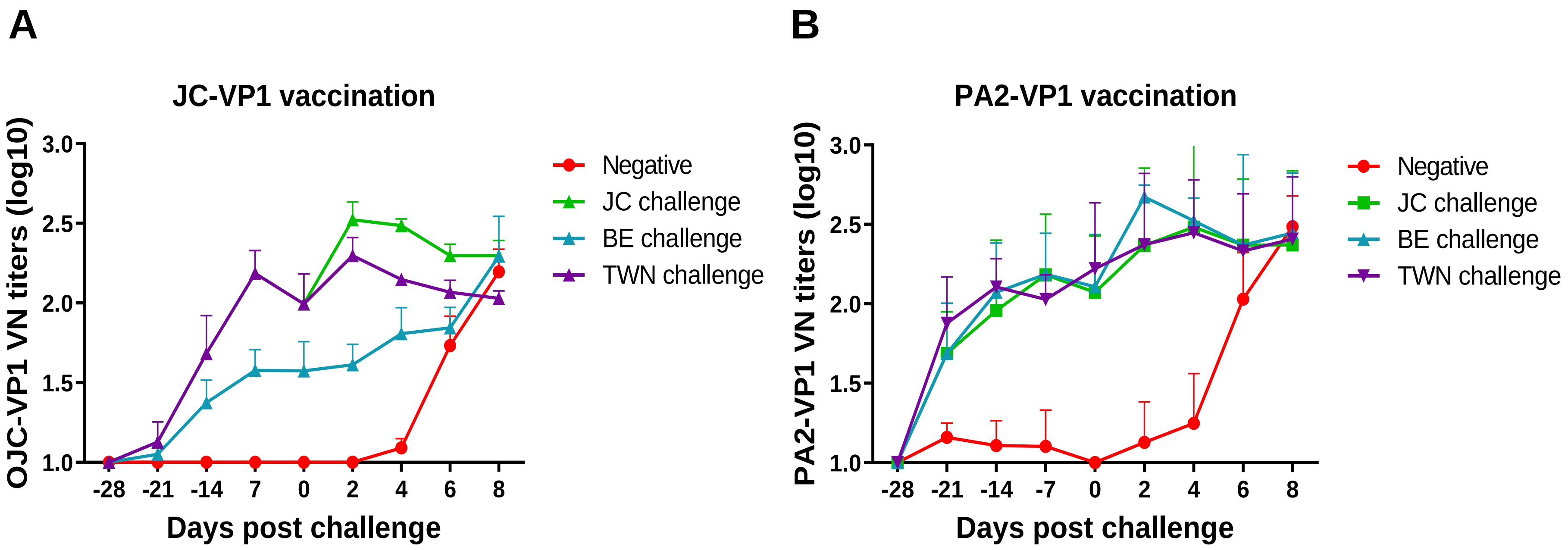

Figure S5. Variation of virus neutralizing titers against vaccine strain after each three topotype challenge in pigs vaccinated with JC-VP1 and PA2-VP1.

(A) Virus neutralizing titers against JC-VP1

(B) Virus neutralizing titers against PA2-VP1
